# Induction of Nickel Accumulation in Response to Zinc Deficiency in *Arabidopsis thaliana*

**DOI:** 10.3390/ijms16059420

**Published:** 2015-04-27

**Authors:** Sho Nishida, Aki Kato, Chisato Tsuzuki, Junko Yoshida, Takafumi Mizuno

**Affiliations:** 1 Graduate School of Bioresources, Mie University, Kurimamachiya-cho 1577, Tsu, Mie 514-8507, Japan; E-Mail: sho3star@gmail.com; 2 Faculty of Bioresources, Mie University, Kurimamachiya-cho 1577, Tsu, Mie 514-8507, Japan; E-Mails: aiko-pinchichance@ezweb.ne.jp (A.K.); chanchi-091122@docomo.ne.jp (C.T.); 512m105@m.mie-u.ac.jp (J.Y.)

**Keywords:** *Arabidopsis thaliana*, nickel, transporter, zinc

## Abstract

Excessive accumulation of nickel (Ni) can be toxic to plants. In *Arabidopsis thaliana*, the Fe^2+^ transporter, iron (Fe)-regulated transporter1 (IRT1), mediates Fe uptake and also implicates in Ni^2+^ uptake at roots; however, the underlying mechanism of Ni^2+^ uptake and accumulation remains unelucidated. In the present study, we found that zinc (Zn) deficient conditions resulted in increased accumulation of Ni in plants, particularly in roots, in *A. thaliana*. In order to elucidate the underlying mechanisms of Ni uptake correlating zinc condition, we traced ^63^Ni isotope in response to Zn and found that (i) Zn deficiency induces short-term Ni^2+^ absorption and (ii) Zn^2+^ inhibits Ni^2+^ uptake, suggesting competitive uptake between Ni and Zn. Furthermore, the Zrt/Irt-like protein 3 (*ZIP3*)-defective mutant with an elevated Zn-deficient response exhibited higher Ni accumulation than the wild type, further supporting that the response to Zn deficiency induces Ni accumulation. Previously, expression profile study demonstrated that *IRT1* expression is not inducible by Zn deficiency. In the present study, we found increased Ni accumulation in *IRT1*-null mutant under Zn deficiency in agar culture. These suggest that Zn deficiency induces Ni accumulation in an *IRT1*-independen manner. The present study revealed that Ni accumulation is inducible in response to Zn deficiency, which may be attributable to a Zn uptake transporter induced by Zn deficiency.

## 1. Introduction

Nickel (Ni) is known to be an essential nutrient for higher plants [1,2]; however, excessive amounts of Ni can be toxic [3]. In Ni-contaminated areas, agricultural crops exhibit Ni-induced impairments (e.g., chlorosis and leaf deformities), and the yields are reduced [4,5,6]. Thus, Ni phytotoxicity is one of the problems that limit agricultural production.

To elucidate the mechanism that underlies Ni phytotoxicity, we studied the molecular mechanism of Ni accumulation in plants using *Arabidopsis thaliana* as the model. We demonstrated that excess Ni is absorbed by the iron (Fe) uptake system in *A. thaliana*, which is associated with iron-regulated transporter 1 (IRT1), the primary Fe uptake transporter in roots [7]. Furthermore, we revealed that excess Ni accumulation induces *IRT1* expression, which is associated with a disorder of Fe homeostasis, suggesting that Ni accumulation further accelerates Ni accumulation by IRT1 [8].

In our previous study, Ni accumulation was greatly decreased by defects in *IRT1* under Fe-deficient conditions, whereas under Fe-sufficient conditions the reduction rate fell significantly, yet excess Ni still accumulated in the mutants, which exhibited Ni phytotoxicity [7]. This indicates that an additional pathway participates in the mediation of Ni accumulation in roots. Physiological evidence indicates that Ni^2+^ uptake competes with the magnesium (Mg) divalent ion in algae [9], barley [10], and spinach [11]. Further, members of the *Arabidopsis* MRS/MGT family of Mg^2+^ transporters have been shown to exhibit Ni^2+^ uptake activities in yeast [12,13]. These reports suggest that the Mg^2+^ uptake system is also a pathway for Ni^2+^ uptake. Furthermore, a previous study suggested that Ni^2+^ is absorbed by uptake systems for divalent heavy metal ions (e.g., Zn^2+^, Cu^2+^) in soybean [14]. Several studies have shown that some members of the Zrt/Irt-like protein (ZIP) family, which are the homologs of IRT1, are active in the transport of these heavy metal ions [15,16,17], suggesting that Ni^2+^ is competitively absorbed by the uptake systems of these divalent cations via ZIP transporters other than IRT1.

In the present study, we found that Ni accumulation increased in *A. thaliana* plants under Zn deficiency in hydroponics. A tracer assay revealed that the ^63^Ni absorption activity was higher in Zn-deficient plants than in the Zn-sufficient plants, showing that the Ni^2+^ uptake activity is upregulated by Zn deficiency. A *zip3* mutant with an elevated Zn-deficient response exhibited increased Ni accumulation in roots compared with that in the wild type, possibly suggesting that the Zn-deficient response increases Ni accumulation. Furthermore, previous studies and our data suggest that the induction of Ni accumulation by Zn deficiency is attributable to an unknown molecular mechanism independent of IRT1.

## 2. Results

### 2.1. Ni Accumulation under Zn Deficiency in Hydroponics

**Figure 1:**
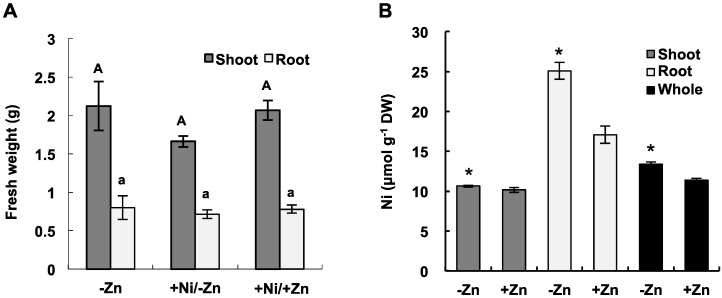
Ni accumulation in plants grown under Zn-sufficient or -deficient conditions. Four-week-old plants were exposed to 25 μmol·L^−1^ NiCl_2_ under Zn-sufficient (5 μmol·L^−1^) or -deficient (0 μmol·L^−1^) conditions for 7 days in a hydroponic culture. (**A**) Fresh weight. The same letters in each series (uppercase for shoots and lowercase for roots) indicate no significant difference (*p* ≥ 0.05, Steel-Dwass test) between treatments; (**B**) Ni concentrations in the shoots, roots, and whole plants. Asterisks denote significant differences (*p* < 0.05, Wilcoxon-Mann-Whitney test) between the Zn-sufficient and -deficient conditions for each tissue. The values represent means ± SD based on four independent experiments.

To investigate the effects of Zn deficiency on Ni accumulation in *A. thaliana*, growth rates and Ni accumulation were determined for 4-week old plants exposed to 25 μmol·L^−1^ NiCl_2_ under Zn-sufficient (5 μmol·L^−1^) or -deficient (0 μmol·L^−1^) conditions for 7 days in a hydroponic culture. As an additional control measure, plants were grown in the Zn-deficient conditions without Ni exposure. There was no statistically significant difference in growth between treatments (Figure 1A). Ni concentrations in the roots were 40% higher under the Zn-deficient conditions than those under the Zn-sufficient conditions (Figure 1B). A slight increase in Ni concentrations was observed in the shoots under the Zn-deficient conditions; however, Ni concentrations in whole plants were significantly higher under the Zn-deficient conditions than under the Zn-sufficient conditions.

### 2.2. Short-Term ^63^Ni Absorption in Zn-Deficient/Sufficient Plants

**Figure 2:**
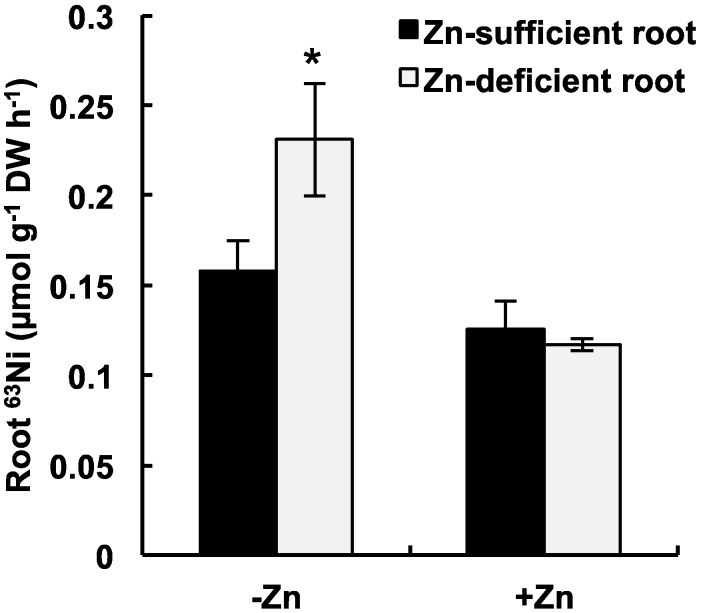
Short-term ^63^Ni uptake by plants. Plants grown under Zn-sufficient (5 μmol·L^−1^) or -deficient (0 μmol·L^−1^) conditions for 4 days were incubated in hydroponic solutions containing 5 μmol·L^−1^ ^63^NiCl_2_ in the presence of Zn (5 μmol·L^−1^ ZnCl_2_) or absence of Zn (0 μmol·L^−1^) for 1 h. Asterisks denote significant differences (*p* < 0.05, Wilcoxon-Mann-Whitney test) between the Zn-sufficient and -deficient roots, and the values represent means ± SD based on four independent experiments.

Ni concentration in roots represents the sum of the amount of Ni absorbed into the cell and the amount of Ni adsorbed onto the cell wall. On the cell wall, metal ions bind loosely to negatively charged sites, and the metals bound to the cell wall are readily removed by washing them with solutions containing competitive cations [11,18,19]. The proportion of Ni^2+^ among the total metal ions in the hydroponic solutions used in the above experiments was 0.0738% in both the Zn-absent and -present conditions. There was probably no difference in the amount of Ni fraction adsorbed in the different Zn conditions; therefore, we assumed that the increased Ni accumulation in roots under the Zn-deficient conditions represented the increased Ni absorption by the cells. Cataldo *et al.* [14] have suggested that Ni^2+^ and Zn^2+^ partially share the same uptake transporter in soybean. This could also be the case in *A. thaliana*, where Ni^2+^ was readily absorbed in the absence of competitive ions in the present study. Moreover, it is possible that the Ni^2+^ uptake activity of the roots was enhanced in response to Zn deficiency. To assess these possibilities, we performed a short-term Ni^2+^ uptake experiment using ^63^Ni-labeled NiCl_2_ (^63^NiCl_2_) and compared difference in Ni^2+^ uptake activities between Zn-deficient and -sufficient plants. One-month-old plants were grown under Zn-deficient (0 μmol·L^−1^) or -sufficient (5 μmol·L^−1^) conditions for 4 days in a hydroponic culture, and ^63^Ni uptake was determined with 5 μmol·L^−1^ ^63^NiCl_2_ in the presence of Zn (5 μmol·L^−1^) or absence of Zn for 1 h. Our preliminary experiments revealed that ^63^Ni was almost undetectable in the shoots using this assay system; therefore, Ni uptake by plants was evaluated on the basis of ^63^Ni accumulation in the roots. To consider only the absorbed ^63^Ni, the apoplastic ^63^Ni was removed by incubating it in a wash solution containing 5 mmol·L^−1^ Ca(NO_3_)_2_ and 5 μmol·L^−1^ cold NiCl_2_, according to the established methods [11,18,19]. Statistical comparisons were made on ^63^Ni accumulation between the Zn-sufficient and -deficient roots in each Zn-present and -absent condition. Under the Zn-absent condition, ^63^Ni accumulation in the Zn-deficient roots was significantly higher (by 1.5-fold) than that in the Zn-sufficient roots (Figure 2), indicating that the ^63^Ni absorption activity was induced in the Zn-deficient roots, as we had expected. But, there was no significant difference in ^63^Ni accumulation between the Zn-sufficient and -deficient roots in the Zn-present condition. The addition of Zn to Zn-present and Zn-absent condition caused a significant decrease in Ni accumulation for both conditions (Wilcoxon-Mann-Whitney test, *p* < 0.05). This is suggesting that Ni^2+^ uptake is competitively inhibited by Zn^2+^, as observed in soybean.

### 2.3. Ni Accumulation in a ZIP3 Mutant

**Figure 3:**
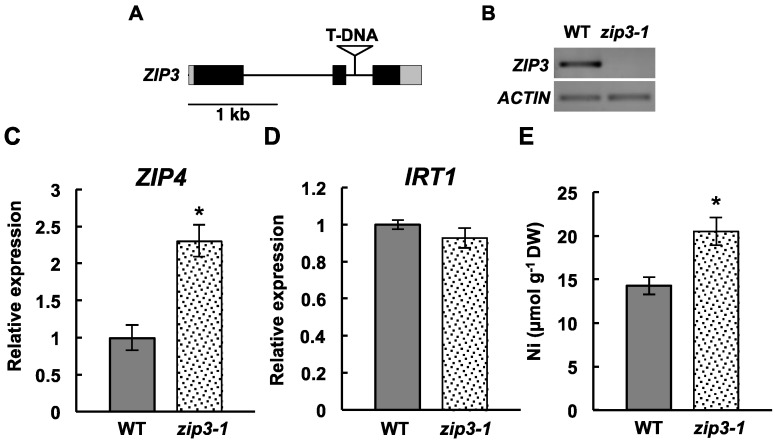
Ni accumulation in the *Zrt/Irt-like protein 3* (*ZIP3*) mutant. (**A**) Scheme showing the T-DNA insertion site in *zip3-1*; (**B**) Semi-quantitative amplification of full-length CDS of *ZIP3* in the roots of the wild type and *zip3-1*. The results shown were obtained after 30 cycles of amplification. *Actin* was amplified as an endogenous control; (**C**,**D**) Quantification of the expression levels of *ZIP4*, a well-known Zn-deficient response indicator and *IRT1* in roots grown in Zn-sufficient condition (5 µM ZnCl_2_) for 1 week. The expression levels relative to *EF1α* are shown; (**E**) Ni accumulation in roots. Four-week-old plants were exposed to 25 μmol·L^−1^ NiCl_2_ under Zn-sufficient conditions (5 μmol·L^−1^ ZnCl_2_) for 1 week in hydroponic culture. The data represent means ± SD based on three (**C**,**D**) and four (**E**) independent experiments. Asterisks denote significant differences (*p* < 0.05, Wilcoxon-Mann-Whitney test) between *zip3-1* and the wild type.

*ZIP3* is a member of the ZIP family, and it has been shown to be highly induced by Zn deficiency in the roots of *A. thaliana* [16,20]. It was also found that *ZIP3* complemented the Zn^2+^-uptake transporter of yeasts [15]. Therefore, it has been suggested that *ZIP3* is involved in Zn nutrition of the roots. We obtained a T-DNA mutant *zip3-1*, which carries a T-DNA insertion in the second intron of *ZIP3* (Figure 3A). The expression of the full length of *ZIP3* was undetectable in *zip3-1* (Figure 3B), confirming that this strain is defective in *ZIP3* expression. Four-week-old plants of the *zip3-1* mutant and wild type were grown under Zn-sufficient conditions (5 µM ZnCl_2_) for 1 week in hydroponics, and we examined *ZIP4* expression, which is a well-known indicator of Zn deficiency [21]. *ZIP4* expression increased more than twofold in *zip3-1* compared with that in Col-0 (Figure 3C), indicating that the Zn-deficient response was elevated in *zip3-*1, even under Zn-sufficient conditions. There was no significant difference in *IRT1* expression between the *zip3-1* mutant and the wild type (Figure 3D). Next, we exposed 4-week-old *zip3-1* mutant plants and wild type plants to 25 µM NiCl_2_ under Zn-sufficient conditions (5 µM ZnCl_2_) for 1 week and determined Ni accumulation in the roots. Ni accumulation increased by 1.5 fold in *zip3-1* compared with that in the wild type (Figure 3E). This result is suggesting that Ni accumulation can be increased in a mutant that exhibits an endogenously elevated Zn-deficient response, which is supporting the hypothesis that Ni accumulation increases in plants in response to Zn deficiency.

### 2.4. Ni Accumulation in the IRT1 Mutant

**Figure 4:**
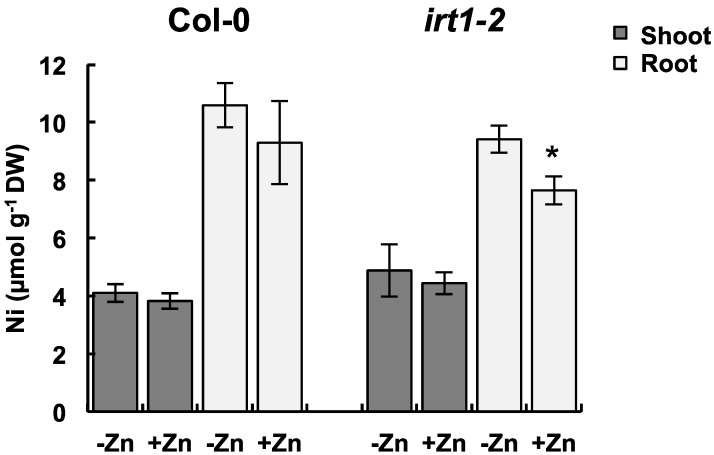
Ni accumulation in *iron-regulated transporter1-2* (*irt1-2*) under Zn-sufficient and -deficient conditions. One-week-old plants were grown on MGRL plates containing 25 μmol·L^−1^ NiCl_2_ and 0 or 5 μmol·L^−1^ ZnCl_2_ for 1 week. Asterisks denote significant differences (*p* < 0.05, Wilcoxon-Mann-Whitney test) between the Zn-sufficient and -deficient conditions. The values represent means ± SD based on four independent experiments.

We also examined Ni accumulation in Zn-deficient and -sufficient conditions in an *IRT1*-defective mutant. We reported two independent *IRT1*-defective mutants in the Col-0 background [7]. But, here we used *irt1-2*, which has a null mutation in *IRT1*, to exclude the effect of IRT1 as much as possible. *irt1-2* carries a T-DNA insertion in the open-reading frame of *IRT1* and expresses a mutated *IRT1* coding for a non-functional form [7]. Because the *irt1-2* plants cannot grow in the normal hydroponic culture, we needed to use an agar plate containing sucrose to maintain *irt1-2* growth as described in our previous report. As a result of preliminary test with Col-0, we confirmed that Zn accumulations in the shoots and roots of plants grown on the Zn-deficient plate were significantly decreased compared with those of plants grown on the Zn-sufficient plate (Figure S1). In Col-0 plants, the mean Ni concentration in roots was increased by 15% in the Zn-deficient conditions compared with that in the Zn-sufficient conditions, although not statistically significant (*p* = 0.3, Wilcoxon-Mann-Whitney test) (Figure 4), possibly suggesting that Ni accumulation is increased by Zn deficiency in agar culture. In *irt1-2* plants, Ni accumulation in roots was significantly increased under the Zn-deficient conditions. These results indicate that the increased Ni accumulation by Zn deficiency is also observed in agar culture at least in *irt1-2*.

## 3. Discussion

In this study, we showed that Ni accumulation was increased by Zn deficiency in *A. thaliana*. An increased Ni accumulation was clearly observed in the roots, whereas the increase was quite small in the shoots. In a previous study, no marked difference was noted in Ni accumulation in the shoots of the *irt1* mutant and the wild type, although Ni accumulation in roots was much lower in *irt1* [7]. Thus, it appears that the effect of increased or decreased Ni absorption in roots is not reflected by Ni accumulation in shoots. Therefore, we mainly focused on Ni accumulation in roots in the present study.

In a radioisotope tracer experiment, we assessed Ni^2+^ absorption in roots treated under Zn-deficient and -sufficient conditions and found that ^63^Ni absorption was higher in Zn-deficient roots, indicating that the Ni^2+^ absorption activity is induced in response to Zn deficiency in wild type plants. We also found that the *zip3* mutant, which exhibits a constitutively elevated Zn-deficient response, accumulated higher levels of Ni than the wild type. This observation further supports that Ni^2+^ absorption is inducible in response to Zn deficiency. In the tracer assay, we also observed that the increased ^63^Ni absorption in Zn-deficient roots was considerably inhibited by adding Zn to the uptake solution, suggesting that Ni^2+^ can be competitively absorbed via a Zn^2+^ uptake pathway, which is similar to that found in soybean [14]. These findings may indicate that Ni^2+^ is absorbed via a Zn^2+^ uptake transporter induced by Zn deficiency in roots.

It is known that IRT1 is active in Zn^2+^ uptake [22,23]; however, its expression is not induced by Zn deficiency [16,20]. Further, the *irt1* mutant exhibited an increased Ni accumulation under Zn-deficient conditions in agar culture in the present study. These are suggesting that the increased Ni accumulation caused by Zn deficiency occurs independently of IRT1. But, we note that further studies are necessary in order to conclude that Zn-deficient induced Ni accumulation observed in hydroponic culture is also independent of IRT1. *IRT1* expression did not increase in the *zip3* mutant, and thus IRT1 was probably not the cause of the increased Ni accumulation in the *zip3* mutant.

The findings of the present study provide considerable insights to elucidate the molecular mechanisms of Ni accumulation in plants. In *A. thaliana*, it has been shown that two closely related members of the basic-region leucine-zipper family of transcription factors, bZIP19 and bZIP23, are responsible for the primary responses to Zn deficiency and that the *bzip19 bzip23* double mutant is unable to induce some genes responsive to Zn deficiency [21]. In the present study, Ni accumulation caused by Zn deficiency could have been controlled by these transcription factors. Our data also suggest that Ni^2+^ is competitively taken up by a Zn^2+^ transporter. The transporter genes that play a role in Zn^2+^ uptake by *A. thaliana* roots are uncertain; however, as previously mentioned, several studies have shown that some ZIP family genes encode Zn^2+^ uptake transporters, which are induced by Zn deficiency [17,20,21]. Therefore, it is expected that other ZIP members will also have Ni^2+^ transport activities. Currently, we are investigating the possible involvement of other *Arabidopsis* ZIP genes in Ni^2+^ uptake.

## 4. Experimental Section

### 4.1. Analysis of Ni Accumulation in Plants Grown in Hydroponic Culture and Agar Plate Culture

The *A. thaliana* ecotype Col-0 was cultured in a hydroponic system, as described in our previous study [7]. Four-week-old plants were irrigated with a hydroponic solution containing 25 μmol·L^−1^ NiCl_2_ and 0 or 5 μmol·L^−1^ ZnCl_2_ and were cultured for 1 week. After exposure, the plants were rinsed with deionized water, and the fresh weights were determined. The plant parts were then dried at 70 °C for 3 days to determine the dry weight. We confirmed that the abundance of free Ni^2+^ in the hydroponic solution was >99% of the total Ni in both the Zn-sufficient and -deficient conditions using GEOCHEM-EZ [24].

Plants with *irt1-2* (SALK_054554), which is a previously described null mutant of *IRT1* in the Col-0 background [7], and the wild type were grown on MGRL-base agar plates, which comprised half-strength MGRL supplemented with 1.5% sucrose, 1.2% ultra-pure agar (Sigma-Aldrich Co., Tokyo, Japan), 5 mmol·L^−1^ MES (pH 5.5), and 50 μmol·L^−1^ FeNa-EDTA. One-week-old seedlings were transferred to agar plates, which were supplemented with 25 μmol·L^−1^ NiCl_2_ and 0 or 5 μmol·L^−1^ ZnCl_2_ and grown for 1 week. After exposure, the plants were sampled as described above.

The plant samples were digested with HNO_3_ and H_2_O_2_ in a heat block. The elemental analysis was performed using an inductively coupled plasma (ICP) atomic emission spectrometer (ICPS-7500; Shimadzu Co., Kyoto, Japan) or ICP mass spectrometer (SPQ9700; Hitachi High-Teck Science Co., Tokyo, Japan).

### 4.2. Short-Term Ni^2+^ Uptake Assay Using ^63^Ni Isotope

One-month-old plants cultured under hydroponic conditions were grown in hydroponic solutions containing 0 or 5 μmol·L^−1^ ZnCl_2_ for 4 days. Next, the roots were rinsed with deionized water and gently blotted, and the plants were irrigated with a hydroponic solution containing 5 μmol·L^−1^ ^63^NiCl_2_ (2.16 µCi·mL^−1^, Nuclitec GmbH, Braunschweig, Germany) with or without 5 μmol·L^−1^ ZnCl_2_, and incubated in the growth chamber under the conditions described above. After 1 h, the plants were placed into wash solutions containing 5 mmol·L^−1^ Ca(NO_3_)_2_ and 5 μmol·L^−1^ NiCl_2_ for 30 min at room temperature. This step facilitated the desorption of ^63^Ni ions, which were adsorbed onto the root cell walls via the equilibrium effect, as previously described [11,18,19]. The ^63^Ni contents of the roots were determined using previously described procedures [7] with a liquid scintillation counter (LSC-5100; Hitachi Aloka Medical Ltd., Tokyo, Japan). The hydroponic culture conditions were the same as those described above.

### 4.3. Identification of the ZIP3-Defective Mutant and Gene Expression Analysis

The T-DNA mutant lines *zip3-1* (CS870394) were acquired from the Syngenta *Arabidopsis* Insertion Library (SAIL) collection via the *Arabidopsis* Biological Resource Center (ABRC). Homozygous insertion mutants were identified using genomic polymerase chain reaction (PCR) with the following primers: 5'-TGA CTA ACA GAA CTA TAG AAG ACG CAT G-3' and 5'-CAT GCC ATT TAT TGT CGA TGA TGA CGA-3'.

The Col-0 and *zip3-1* plants were grown in hydroponics for 4 weeks, as described above, and the plants were then transferred to Zn-sufficient conditions (5 μmol·L^−1^ ZnCl_2_) and cultured for 1 week. Total RNA was extracted from the roots, according to the method reported by Suzuki *et al.* [25]. Genomic DNA in the extracts was digested with DNase I (Takara Bio Inc., Shiga, Japan), and the first-strand cDNA was synthesized from the total RNA using the PrimeScript™ RT reagent kit (Takara Bio Inc.). The cDNA was used in the following semi-quantitative and quantitative RT-PCR assays.

Amplification of the full-length coding region of *ZIP3* (AT2G32270) was conducted using the above mentioned gene-specific primers and cDNA corresponding to 5 ng of RNA. PrimerSTAR^®^ HS DNA Polymerase (Takara Bio Inc.) was used for PCR. *ACTIN* (AT2G37620) was also amplified as an internal control with the following primers: 5'-AAT TGG GAT GAC ATG GAG AAG ATT TGG-3' and 5'-TGG AGT TAT AGG TGG TTT CAT GGA TAC-3'. The PCR products on the agarose gels were detected by staining with ethidium bromide.

cDNA corresponding to 5 ng of RNA was used for quantitative RT-PCR with SYBR Premix Ex Taq II (Takara Bio Inc.). The PCR reaction was performed with a Thermal Cycler Dice Real Time System II^®^ (Takara Bio Inc.). For the thermal profile, instructions included in the manual provided with the PCR kit were followed. The specific primers used for *ZIP4* (AT1G10970) were as follows: 5'-GGT TGT ATC CTC CAG GCT GAG T-3' and 5'-TGG TGT TGT TAC CGC GAA AA-3'. The specificities of the primers were confirmed by analyzing the dissociation curves and agarose gel electrophoresis of the PCR products. The specific primers for *EF1*α designed by Takano *et al.* [26] were used.

## 5. Conclusions

In the present study, we demonstrated that Zn deficiency induces Ni absorption activity in roots and Zn^2+^ inhibits Ni^2+^ uptake. We also found Ni accumulation can be increased in *IRT1*-null mutant. Taken together, our data suggest that Ni is absorbed by an unknown Zn transporter induced by Zn deficiency.

## Supplementary Materials

Supplementary materials can be found at http://www.mdpi.com/1422-0067/16/05/9420/s1.
