# Supplementary material for: Induction of Nickel Accumulation in Response to Zinc Deficiency in Arabidopsis thaliana
Source: Int J Mol Sci. 2015 Apr 27;16(5):9420–30. doi: 10.3390/ijms16059420 (PMC4463596; doi:10.3390/ijms16059420)
Supplement: Supplementary file 1 [file ijms-16-09420-s001.pdf]

Supplementary Information

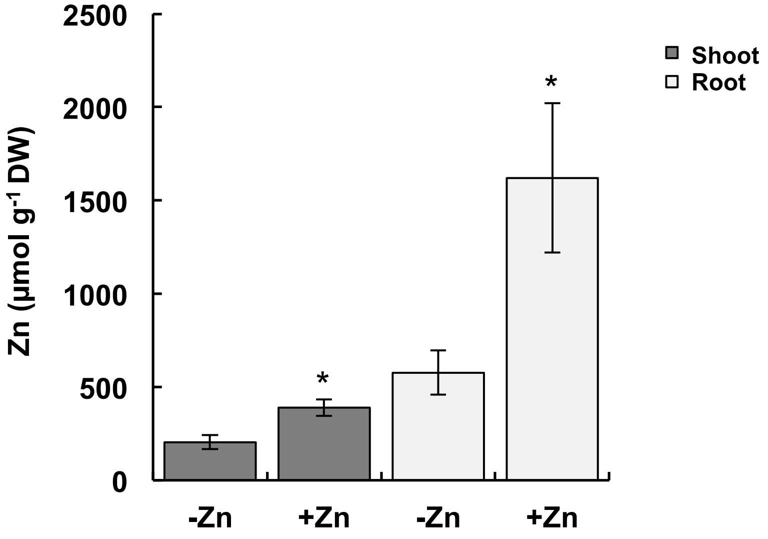

Figure S1. Ni accumulation in the plants of Col-0 grown on Zn-sufficient and -deficient plates.

One-week-old plants were grown on MGRL plates containing 0 or 5 $\mu mol \cdot L^{-1}$ $ZnCl_2$ for 1 week. Asterisks denote significant differences ($p < 0.05$, Wilcoxon-Mann-Whitney test) between the Zn-sufficient and -deficient conditions. The values represent means ± SD based on four independent experiments.
